# Esketamine use in real-world clinical practice in patients with treatment-resistant depression

**DOI:** 10.1192/j.eurpsy.2025.10096

**Published:** 2025-08-26

**Authors:** Ismael Conejero, Raquel Alvarez García, Alejandro Porras-Segovia, Ana María De Granda Beltrán, Sergio Benavente López, Ezequiel Di Stasio, Lucía Albarracín-García, Jorge Lopez-Castroman, Philippe Courtet, Enrique Baca-Garcia, Memind Group

**Affiliations:** 1 Department of Psychiatry, Hospital Universitario Fundación Jiménez Díaz, Instituto de Investigación Sanitaria Fundación Jiménez Díaz, Madrid, Spain; 2 Departamento de Psiquiatría, Universidad Autónoma de Madrid, Madrid, Spain; 3 Department of Psychiatry, Hospital Universitario Fundación Jiménez Díaz, Madrid, Spain; 4 Department of Psychiatry, CHU Nîmes, IGF, INSERM, University of Montpellier, Nîmes, France; 5 Departamento de Psiquiatría, Hospital Rey Juan Carlos Móstoles, Madrid, Spain; 6 Departamento de Psiquiatría, Hospital Universitario Infanta Elena Valdemoro, Madrid, Spain; 7 Departamento de Psiquiatría, Hospital Universitario General de Villalba, Madrid, Spain; 8 Department of Psychiatry, Radiology, Public Health, Nursing and Medicine, University of Santiago de Compostela, A Coruña, Spain; 9 Department of Emergency Psychiatry and Acute Care, Centre Hospitalier Universitaire Montpellier, University of Montpellier, Montpellier, France; 10 Department of Psychology, Universidad Católica del Maule, Talca, Chile; 11 Centro de Investigación Biomódica en Red de Salud Mental (CIBERSAM), Research Group CB/07/09/0025, Madrid, Spain

**Keywords:** augmentation treatment, esketamine, naturalistic study, treatment resistant depression

## Abstract

**Background:**

Esketamine has been shown to produce a major antidepressant response in patients with treatment-resistant depression (TRD). We evaluated the factors associated with achieving remission in these individuals.

**Methods:**

The study was carried out across four psychiatry departments in Madrid, Spain. Patients aged over 18 years were included if they received esketamine as an augmentation treatment for TRD. Standard esketamine protocol included an induction phase (4 weeks) and a maintenance phase (5 to 8 weeks). Subsequent treatment continuation was proposed. Clinical data and scores at the Clinical Global Impression scales were measured following each esketamine administration.

**Results:**

Sixty-five patients initiated the treatment, and 45 patients (69.2%) completed the standard protocol. The median number of esketamine administrations was 19. The mean age was 53.09 and 52.3% of the patients were females. Out of the whole sample, 36 (55%) of the patients achieved remission over the follow-up. Remission rates elevated to 67% in those who completed the standard protocol, and to 70% in those having received more than 19 esketamine administrations. Achieving remission over the follow-up was associated with the absence of dissociative symptoms, and with completing the standard esketamine protocol (OR = 0.229, p = 0.045; and OR = 4.538, p = 0.025, respectively). Receiving more than 19 esketamine administrations was associated with remission over the follow-up (OR = 6.513, p = 0.006).

**Conclusions:**

Our results suggest that extending the numbers of esketamine administration may increase the chances to obtain remission. Adverse effects did not impact the treatment course.

## Introduction

Depression is a common mental disorder with the highest prevalence in Europe reaching 11.32% lifetime, and 10.3% in the Spanish population [1]. While Major Depressive Disorder (MDD) has been often considered as a transient condition, epidemiological data suggest that more than 30% of depressed individuals do not achieve remission following antidepressant treatment [2, 3]. In addition, MDD is a risk factor for suicide with a relative risk exceeding 7.6 compared to general population [4], especially in individuals suffering from treatment-resistant depression (TRD) [5]. Despite the lack of consensus, the European Medicines Agency and the US Food and Drug Administration defined TRD in patients failing to achieve response to at least two antidepressant trials of different classes despite adequate dose, duration and adherence to treatment [6]. Patients with TRD show lower quality of life, as well as increased work disability and utilization of health resources.

The clinical guidelines offer a range of recommendations for the treatment of TRD, including psychotherapeutic approaches, pharmacological alternatives, and especially the combination of both. Treatments currently available have limited effectiveness and clinical response is often delayed. Intranasal esketamine (Spravato®), a new antidepressant treatment increases response rates in patients with a TRD [7]. In 2022, the Spanish Agency for Medicines and Health Products (AEMPS) authorized esketamine in combination with an oral antidepressant drug for the treatment of TRD in adults who did not respond to at least two different antidepressant treatments [8].

Trials have evidenced rapid efficacy of esketamine as an augmentation treatment for major depression [9, 10], and also as a treatment for TRD [11]. In particular, the results of a prior real-world Italian study supported the safety and tolerability of the treatment [12], and showed a response rate of 76% at 6 months to follow-up [13]. Furthermore, esketamine showed effectiveness in various patients subsamples, including individuals with bipolar TRD [14] and in those with comorbid substance use disorders [15]. The combination of esketamine with vortioxetine was linked with an increased reduction of emotional blunting and better tolerance as compared with other antidepressant augmentation strategies [16]. Younger age, being employed and low number of prior antidepressant trials were identified as predictors of response and remission over one month [17]. However, its therapeutic effect and long-term safety need to be further investigated.

While the response to antidepressants is measured through the Montgomery-Åsberg Depression Rating Scale (MADRS) in research [9, 18], clinical changes in patients receiving treatment for TRD in routine care are easy to investigate using the Clinical Global Impressions-Severity (CGI-S) that assesses severity at the time of the evaluation, and using the Clinical Global Impressions-Improvement scales (CGI-I) which investigates the evolution of the severity since last visit [19].

Hence, in this real-world naturalistic cohort study we aimed at evaluating the factors associated with achieving remission during esketamine augmentation treatment measured through the CGI-S and through the CGI-I. We also assessed the factors associated with the premature interruption of treatment in patients with TRD.

## Materials and methods

### Setting and design

This observational study was carried out across four psychiatry departments: Rey Juan Carlos University Hospital, Fundación Jiménez Díaz, Infanta Elena Hospital, and General University Hospital of Villalba. All of them are part of Spain’s National Health Services and affiliated with the Fundación Jiménez Díaz. The study was approved by the Hospital Fundación Jiménez Díaz Ethics Committee (CEIm-FJD) on 18th March 2024, and patients’ information was handled as stated in Spanish and European regulations on data protection and patients’ digital rights. All the participants provided written informed consent before entering the study.

### Sample

The patients aged 18 years and older were included in the analysis if they filled the European Medicine Agency’s criteria for a diagnosis of TRD – i.e failure to achieve response to at least two prior antidepressant trials despite adequate dose, duration and adherence to treatment – [6, 20], and if they received esketamine as an augmentation treatment for TRD. The use of esketamine in routine care started in January 2023 in the four centres. Patients with a TRD were proposed intranasal administration of flexible doses of esketamine (28 mg, 56 mg or 84 mg/administration) according to clinical judgment for augmenting antidepressant treatment according to the following sequences:

- Standard esketamine protocol including an induction phase (2 administrations per week for 4 weeks, 8 in total), and a maintenance phase (1 administration per week for 5–8 weeks, 5–8 in total).
- Treatment continuation (1 weekly administration or every 2 weeks) according to clinician’s judgment and patient’s choice.

All the patients received concomitant standard clinical care for TRD involving at least one antidepressant (Selective Serotonin Reuptake Inhibitor/ Serotonine-Norepinephrine Reuptake Inhibitor or other antidepressant), and eventual psychotherapy as recommanded [6]. Current antidepressant medication was unchanged at esketamine initiation.

The routine sociodemographic and clinical data from 75 patients recorded in the electronic medical records at baseline and after each esketamine administration were anonymized and extracted in compliance with Spanish laws on the Protection of Personal Data.

### Variables and measures

The following information was drawn from structured fields in electronic health records: baseline sociodemographic data, psychiatric diagnosis, comorbidities and depression subtype according to the International Statistical Classification of Diseases and Related Health Problems 10th (ICD-10) criteria (single episode (F32.x), or recurrent depressive disorder (F33.x)). After each esketamine administration, clinicians assessed the occurrence of suicidal ideation and attempts, treatment dosage, and the CGI level. The occurrence of dissociative symptoms, headache, hypertension, anxiety and drowsiness were systematically assessed after treatment intake. The CGI-S scale includes items concerning psychosocial aspects, behavior, symptoms and the impact of depression on daily life. It allows to establish different levels of disease based on the physician’s clinical experience. The CGI-I is measured by comparing the patient’s baseline symptom severity with its state following treatment administration. According to Morrens et al (2022), remission was defined in patients with a decrease of ≥2 points from the baseline CGI-S value or a CGI-S score of ≤3 points over the follow-up (from slightly depressed to normal) [19].

During the last visit of the follow-up, physicians assessed the general clinical improvement. All the data were retrieved from the electronic health records (HER).

### Statistical analysis

All statistical analyses were performed using the Statistical Package for the Social Sciences (SPSS) version 29. First, we provided descriptive statistics of the whole population and performed univariate analyses to compare the clinical characteristics of patients receiving esketamine according to the occurrence of remission and premature interruption of treatment. The univariate analyses were performed using F-exact test, chi-square, or ANOVA.

Then we used logistic regression models to assess the multivariate relationship between the outcomes (the occurrence of remission and premature interruption of treatment), and the clinical factors associated in the univariate analysis with p < 0.1. Overall, the significance level was set at p < 0.05, using 2-sided tests and 95% confidence intervals.

## Results

### Baseline characteristics of the sample

**Figure 1.**
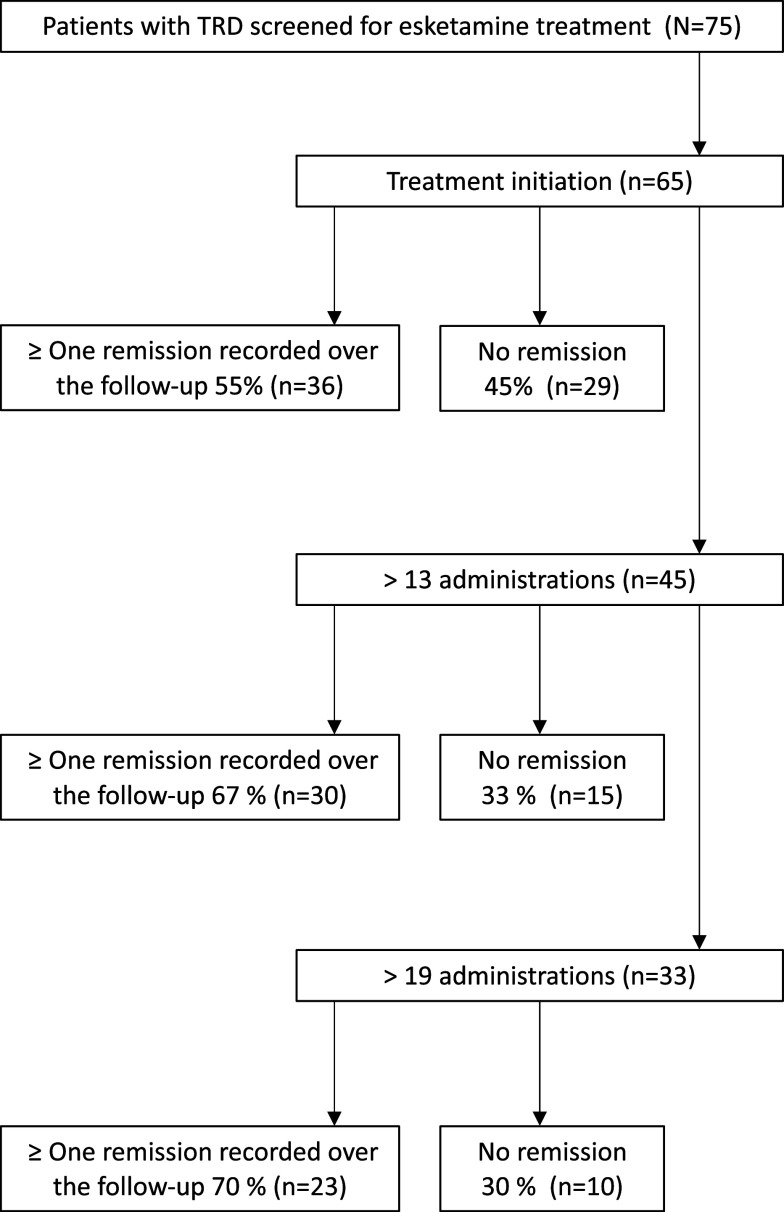
Flow chart.

Out of the 75 candidate patients screened for esketamine, 65 initiated the treatment and were evaluated through the CGI measures over the standard protocol. Out of them, 45 patients (69.2%) completed the standard protocol (i.e., received esketamine at least during the induction and the 5 weeks maintenance phases). The median number of esketamine administrations per subject was 19. The mean esketamine dosage was 66.04 mg [SD = 15.39] per administration in the patient sample. The Figure 1 shows the study flow chart.

The baseline clinical characteristics of the patients are reported in Table 1. The mean age of the sample was 53.09 [SD = 10.15] and 52.3% of the patients were females (N = 34). The mean number of esketamine administrations was 20.42 [SD = 14.60]. Overall, 4.6% of the patients were diagnosed with bipolar disorder, 60% had recurrent depressive disorder, 12.3% were diagnosed with comorbid substance use disorder, 36.9% with a comorbid personality disorder, and 58.5% with a comorbid anxiety disorder. Over the follow-up, 32.3% reported suicidal ideation. In terms of esketamine-related adverse effects, 26.6% of patients experienced a dissociative episode, 9.4% reported headaches, 21.9% had hypertensive episodes, and 10.9% experienced drowsiness.

**Table 1.** Occurrence of remission recorded with CGI-S over the follow-up according to the clinical characteristics at baseline and to the number of treatment administration^a^

Clinical characteristics^b^	Whole sample (n = 65)	≥ One remission (n = 36)	No remission (n = 29)	Significance (p-value)
Age	53.09 [10.15]	53.83 [10.17]	51.93 [11.18]	0.476
<50	21 (32.3%)	10 (27.8%)	11 (37.9%)	0.384
>50	44 (67.7%)	26 (72.2%)	18 (62.1%)	
Gender (female)				
Female	34 (52.3%)	21 (58.3%)	13 (44.8%)	0.279
Male	31 (47.7%)	15 (41.7%)	16 (55.2%)	
Number of administrations	20.42 [14.60]	25.11 [16.46]	14.97 [9.65]	0.005
Number of esketamine administrations (median number in whole sample = 19)				
<19	32 (49.2%)	13 (36.1%)	19 (65.5%)	0.018
>19	33 (50.8%)	23 (63.9%)	10 (34.5%)	
Number of esketamine administration (standard protocol =13)				
<13	20 (30.8%)	6 (16.7%)	14 (48.3%)	0.006
>13	45 (69.2%)	30 (83.3%)	15 (51.7%)	
Mean Esketamine dosage	66.04 [15.39]	65.03 [16.68]	67.28 [13.81]	0.562
Dissociative episode during treatment (missing = 1)	17 (26.6%)	5 (13.9%)	12 (41.4%)	0.015
Psychiatric comorbidities				
Bipolar disorder	3 (4.6%)	1 (2.8%)	2 (6.9%)	0.431
Recurrent depression (F33)	39 (60%)	21 (58.3%)	18 (62.1%)	0.760
Substance use disorder (F10.)	8 (12.3%)	5 (13.9%)	3 (10.3%)	0.665
Personality disorder (F60.)	24 (36.9%)	14 (38.9%)	10 (34.5%)	0.714
Anxiety disorders (F40.)	38 (58.5%)	23 (63.9%)	15 (51.7%)	0.323
Suicidal ideation	21 (32.3%)	12 (33.3%)	9 (31%)	0.844
Treatment adverse effects				
Headache	6 (9.4%)	3 (8.6%)	3 (10.3%)	0.809
Hypertensive episodes	14 (21.9%)	7 (20%)	7 (24.1%)	0.690
Anxiety	9 (14.1%)	6 (17.1%)	3 (10.3%)	0.436
Drowsiness	7 (10.9%)	5 (14.3%)	2 (6.9%)	0.346

a Remission is defined according to Morrens Criteria: CGI-S < 4 or CGI-S decrease ≥2 from baseline.

b Data are means [Standard Deviation], or number (%).

### Factors associated with achieving remission

**Figure 2.**
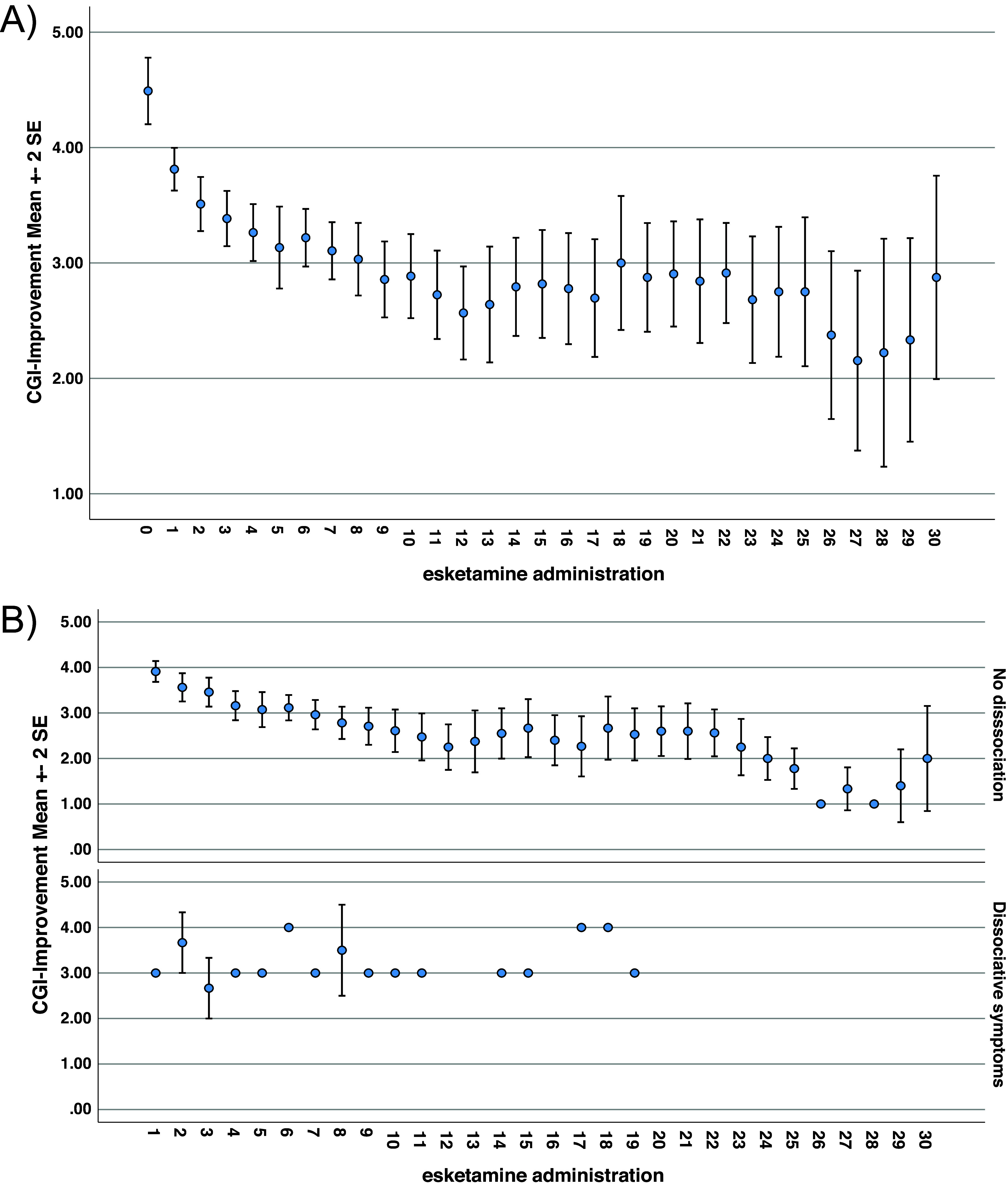
(A) Clinical improvement measured through the CGI-I scale over the follow-up. (B) Clinical improvement measured through the CGI-I scale over the follow-up according to the occurrence of dissociative symptoms subsequently to esketamine intake.

Remission was assessed using CGI-S measurement and defined according to Morrens’ criteria at each clinical visit. Out of the whole sample, 36 (55%) of the patients achieved remission over the follow-up. Overall remission rates elevated to 67% in those who completed the standard protocol, and to 70% in those having received more than 19 esketamine administrations, (i.e., the median number; Figure 1). Figure 2A shows the clinical improvement according to the CGI-I scale over the follow-up.

The occurrence of remission measured during follow-up was significantly associated with a higher mean number of esketamine administration (25.11 [SD = 16.46] vs 14.97 [SD = 9.65] in patients remitted and not remitted respectively, p = 0.005); with completing the standard esketamine protocol (30 (83.3%) vs 15 (51.7%) in patients remitted and not remitted respectively, p = 0.006); with a number of administration greater than 19 (23 (63.9%) vs 10 (34.5%) in patients remitted and not remitted respectively, p = 0.018); and was less frequent when dissociative symptoms occurred during esketamine administration (5 (13.9%) vs 12 (41.4%) in patients remitted and not remitted respectively, p = 0.015). The results are shown in Table 1. Figure 2B highlights clinical improvement measured through the CGI-I scale over the follow-up according to the occurrence of dissociative symptoms subsequently to esketamine intake.

In the multivariate analysis taking into account possible confounding factors, the occurrence of remission over the follow-up was associated with the absence of dissociative symptoms during esketamine administration, and with completing the standard esketamine protocol (OR = 0.229 95%CI [0.054–0.966], p = 0.045; and OR = 4.538 95%CI [1.213–16.974], p = 0.025 in model 1, respectively). The results are shown in Table 2. In model 2, receiving more than 19 esketamine administrations was associated with remission over the follow-up (OR = 6.513 95%CI [1.693–25.060], p = 0.006).

**Table 2.** Occurrence of remission over the follow-up according to the clinical characteristics and to the number of esketamine administration^a^

		≥ One remission				
		Model 1		Model 2		
Clinical characteristics	Estimated odds ratio (exp(b))^b^	95% CI for Exp(b)	Significance (p-value)	Estimated odds ratio (exp(b))^b^	95% CI for Exp(b)	Significance (p-value)
Age > 50 (years)	1.167	0.305–4.470	0.821	1.475	0.369–5.887	0.582
Gender (male)	0.378	0.084–1.697	0.204	0.394	0.088–1.763	0.223
Dissociative episode during treatment	0.229	0.054–0.966	0.045	0.131	0.027–0.621	0.011
Center^c^						
HRJC	0.421	0.042–4.252	0.464	0.686	0.078–6.036	0.734
HIE	0.305	0.052–1.769	0.185	0.198	0.030–1.303	0.092
HGV	2.865	0.471–17.430	0.253	3.380	0.512–22.304	0.206
Number of Esketamine administration > 13	4.538	1.213–16.974	0.025	–	–	–
Number of Esketamine administration > 19 (median)	–	–	–	6.513	1.693–25.060	0.006

a Data are means [Standard Deviation], or number (%).

b The association between the clinical characteristics and treatment patterns, and the overall improvement of depression was tested in a logistic regression model. We included Age, gender and center in both models 1 & 2.

c HRJC: Rey Juan Carlos University Hospital; HIE: Infanta Elena Hospital; HGV: General University Hospital of Villalba.

### Factors associated with the number of esketamine administrations

During the treatment course, 20 patients (30.8%) discontinued esketamine before completing the standard protocol (induction and maintenance phases), while 33 patients (50.8%) received more than the median of 19 treatments. In the univariate analysis, drowsiness following esketamine administration was the only factor significantly associated with receiving more than 19 treatments (drowsiness occurred in 21.9% of patients (n = 7) who received more than 19 administrations compared to 0% in those who received fewer than 19, p = 0.005). The results are detailed in Table 3.

**Table 3.** Quantities of esketamine intake according to the clinical characteristics at baseline and to the adverse effects of treatment^a^

Clinical characteristics	Whole sample (n = 65)	≥ 13 Esketamine administrations (Standard protocol, n = 45)	Significance (p-value)	≥ 19 Esketamine administrations (n = 33)	Significance (p-value)
Age	53.09 [10.15]	53.27 [10.99]	0.750	52.64 [10.04]	0.790
<50	21 (32.3%)	13 (28.9%)	0.377	11 (33.3%)	0.857
>50	44 (67.7%)	32 (71.1%)		22 (66.7%)	
Gender (female)					
Female	34 (52.3%)	22 (48.9%)	0.408	16 (48.5%)	0.531
Male	31 (47.7%)	23 (51.1%)		17 (51.5%)	
Psychiatric comorbidities					
Bipolar disorder	3 (4.6%)	2 (4.4%)	0.922	1 (3%)	0.536
Recurrent depression (F33)	39 (60%)	26 (57.8%)	0.583	22 (66.7%)	0.265
Substance use disorder (F10.)	8 (12.3%)	6 (13.3%)	0.706	6 (18.2%)	0.143
Personality disorder (F60.)	24 (36.9%)	18 (40%)	0.441	15 (45.5%)	0.148
Anxiety disorders (F40.)	38 (58.5%)	26 (57.8%)	0.867	21 (63.6%)	0.390
Suicidal ideation	21 (32.3%)	16 (35.6%)	0.401	8 (24.2%)	0.158
Treatment adverse effects (missing = 1)					
Dissociative episode during treatment	17 (26.6%)	10 (22.7%)	0.303	9 (28.1%)	0.777
Cephalalgia	6 (9.4%)	6 (13.6%)	0.083	5 (15.6%)	0.086
HTA	14 (21.9%)	11 (25%)	0.370	8 (25%)	0.545
Anxiety	9 (14.1%)	7 (15.9%)	0.528	7 (21.9%)	0.072
Drowsiness	7 (10.9%)	7 (15.9%)	0.059	7 (21.9%)	0.005

a Data are means [Standard Deviation], or number (%).

In the multivariate analysis taking into account possible confounding factors, this association did not remain significant. Results are not shown.

## Discussion

In this naturalistic retrospective study, most of the patients with TRD receiving esketamine showed remission over the course of the treatment. Remission rates were positively associated with the number of esketamine administrations and with the absence of dissociative symptoms recorded over the follow-up. Receiving more than 19 administrations increased overall chances of remission. Finally, no clinical factors predicted the total quantity of esketamine received.

### Factors associated with esketamine efficacy

Although the placebo controlled Transform 1 clinical trial and the Transform 3 study in older adults failed to show significant effect of esketamine nasal spray as an add-on treatment of TRD [21], Popova et al. (2019) reported efficacy of flexible doses of esketamine against placebo measured through the MADRS score at 4 weeks [22]. Doses varied between 56 and 84 mg. Esketamine effect was predominant in clinical subgroups including females, patients aged between 45 and 64 years, or prior treatment failures ≥3 [22]. Similarly, esketamine was more effective in patients with ≥3 prior antidepressant failures in the Transform 1 trial [21]. Ochs-Ross et al. (2020) reported a significant difference in patients aged between 65 and 74, while not in those aged 75 and over [23].

In our study, the overall remission rate was related with the number of esketamine administrations. While expending the length of esketamine treatment may be associated with higher rates of patients showing remission, longer follow-up may increase chances to observe natural remission of depressive symptoms. Further comparative studies should be conducted to confirm the specific effect of extending esketamine administration upon achieving remission in patients who failed to improve during induction and maintenance phases. Moreover, from the 36 patients who achieved remission over the follow-up, 9 (25%) filled remission criteria at the final evaluation. Hence, maintaining remission remains challenging in individuals with TRD, although other studies showed higher rates of remission maintenance at study endpoints [24, 25]. Other therapeutic strategies involving non-invasive brain stimultation protocols were suggested to help prevent depression relapse [26]. Hence, their efficacy in maintaining remission may be tested whether in combination, or as comparator to esketamine in TRD [27].

In addition, we reported that remission rate was inversely associated with the frequency of dissociative symptoms. A prior systematic review suggested an absence of association between dissociation and antidepressant outcomes in the patients treated with esketamine [28]. Subsequent post-hoc analyses found no relationship between dissociation scores measured with the Clinician Administered Dissociative States Scale (CADSS) and the effect of esketamine in patients with TRD [29, 30]. In a recent study, the intensity of dissociative symptoms explained the antidepressant effect only within the 24 hours subsequent to the administration [31], while this effect occurred within a specific range of CADSS level [31]. Those diverging results may relate to different methodologies regarding dissociation assessment, as in our study the occurrence of dissociative symptoms were recorded according to ICD-10 criteria. Furthermore, the intensity of the dissociative symptoms was not quantified, which prevented us to investigate its relationship with the antidepressant response.

Also, we found no significant relationship between the remission rate and sociodemographic factors including age and gender, although individuals aged over 50 and females tended to be more represented in patients showing remission. The lack of association may be due to limited statistical power.

### Factors associated with completing standard protocol

In our study, 69% of the patients finalized the standard esketamine protocol. Discontinuation rates appear higher than in prior controlled randomized trials [10]. As esketamine intake was proposed as part of routine care, it is likely that the administration protocol may have been applied with increased flexibility. No factors predicted the overall quantities of esketamine administered.

Interestingly, treatment adverse effects were not related with discontinuation rates over the follow-up, which confirms the general favorable tolerability profile reported in prior studies [32]. The most frequent adverse event reported in our real-world study was the occurrence of dissociative symptoms (27%). This is in accordance with rates of dissociative symptoms reported by Popova [16], that reached 26% in the active treatment group [22].

### Limitations and strengths of the study

This study reflects the real-world use of esketamine as an adjunctive treatment for TRD in psychiatric clinics, hence it is limited by several factors. First, although we assessed the remission of depression through Morrens’ criteria involving the measure of CGI-S score [19], our results should be confirmed with other scales such as the MADRS which has been widely validated in depression treatment trials [18]. Second, our study lacked control group. Further comparative studies may help confirm the specific effect of esketamine treatment length upon the remission rates, against the natural evolution of the disease. Nevertheless, as our study was conducted in individuals bearing TRD, depression is unlikely to show spontaneous remission at short or mid-term in our sample. Also, the size of the sample with complete data remains limited as some variables (such as CGI) were not systematically collected by clinicians at all time points. In fact, the post hoc power analysis on the sample size of 65 patients showed a statistical power of 67.7% related to CGI scores. Moreover, interobserver differences may have skewed the results within each psychiatric centres or between hospitals. However, we found no centre effects when performing multivariate analysis. Finally, this study is retrospective with an open label design, and data were retrieved from clinical records. This prevented us from further comparison with other standardized protocols. This also limited the possibility to control results for a wider range of potential confounding factors as they were not all systematically recorded in patient files. While the patients included in the analysis responded to TRD criteria as defined by the European Medicine Agency (that is failure to achieve response to at least two prior antidepressant trials of different classes despite adequate dose, duration and adherence), the exhaustive list of prior administered treatments was not collected in the current study. Hence, we were not able to investigate the impact of the number of prior treatment failure on the patients’ outcomes. Nevertheless, prior real-world studies failed to evidence any effect of the number of antidepressant trials lifetime [12], nor effects of the number of concomitant antidepressant medication [33].

Conversely, our study has several strengths. This is a naturalistic study reflecting real daily clinical practice. Hence, the results are more generalizable since the patient sample was less strictly selected, especially regarding the existence of psychiatric comorbidities. Furthermore, the use of CGI-S as outcome reflects clinical practice due to its large utilization by clinicians. In addition, remission criteria based on CGI-S have been previously validated [19].

## Conclusions

This real-world naturalistic study suggests that adjunctive treatment with intranasal esketamine may be beneficial for most patients with TRD. Our results suggest that the length of esketamine treatment may predict remission rates in TRD. Especially, extending the numbers of esketamine administration may increase the chances to obtain remission. This results should be confirmed in further comparative studies assessing the natural evolution of depression. Furthermore, we found that the adverse effects measured did not significantly impact the treatment course. Hence, the use of esketamine appears well tolerated in routine clinical care with overall favorable outcomes. Those outcomes may be easily assessed through evaluation tools widely used in psychiatric practice. Further naturalistic studies are needed to evaluate esketamine utilization in long term.

## Data Availability

Data will be made available upon request.

## References

[1] Gutiérrez-Rojas L, Porras-Segovia A, Dunne H, Andrade-González N, Cervilla JA. Prevalence and correlates of major depressive disorder: A systematic review. Braz J Psychiatry. 2020;42(6):657–72. 32756809 10.1590/1516-4446-2019-0650 PMC7678895

[2] McAllister-Williams RH. When depression is difficult to treat. Eur Neuropsychopharmacol. 2022;56:89–91. 34991000 10.1016/j.euroneuro.2021.12.007

[3] Rush AJ, Aaronson ST, Demyttenaere K. Difficult-to-treat depression: A clinical and research roadmap for when remission is elusive. Aust N Z J Psychiatry. 2019;53(2):109–18. 10.1177/0004867418808585 30378447

[4] Moitra M, Santomauro D, Degenhardt L, Collins PY, Whiteford H, Vos T, et al. Estimating the risk of suicide associated with mental disorders: A systematic review and meta-regression analysis. J Psychiatr Res. 2021;137:242–9. 33714076 10.1016/j.jpsychires.2021.02.053 PMC8095367

[5] Reutfors J, Andersson TML, Tanskanen A, DiBernardo A, Li G, Brandt L, et al. Risk factors for suicide and suicide attempts among patients with treatment-resistant depression: Nested case-control study. Arch Suicide Res. 2021;25(3):424–38. 31774374 10.1080/13811118.2019.1691692

[6] McIntyre RS, Alsuwaidan M, Baune BT, Berk M, Demyttenaere K, Goldberg JF, et al. Treatment-resistant depression: Definition, prevalence, detection, management, and investigational interventions. World Psychiatry. 2023;22(3):394–412. 37713549 10.1002/wps.21120 PMC10503923

[7] Mahase E. Esketamine is approved in Europe for treating resistant major depressive disorder. BMJ. 2019;367:l7069. 31862692 10.1136/bmj.l7069

[8] Informe de Posicionamiento Terapéutico de esketamina (Spravato®) en trastorno depresivo mayor resistente al tratamiento [Internet]. Agencia Española de Medicamentos y Productos Sanitarios. 2022 [cité 2 août 2024]. Disponible sur: https://www.aemps.gob.es/informa/informes-de-posicionamiento-terapeutico/informe-de-posicionamiento-terapeutico-de-esketamina-spravato-en-trastorno-depresivo-mayor-resistente-al-tratamiento/

[9] Papakostas GI, Salloum NC, Hock RS, Jha MK, Murrough JW, Mathew SJ, et al. Efficacy of Esketamine augmentation in major depressive disorder: A meta-analysis. J Clin Psychiatry. 2020;81(4):6603. 10.4088/JCP.19r12889 32459407

[10] Zheng W, Cai DB, Xiang YQ, Zheng W, Jiang WL, Sim K, et al. Adjunctive intranasal esketamine for major depressive disorder: A systematic review of randomized double-blind controlled-placebo studies. J Affect Disord. 2020;265:63–70. 31957693 10.1016/j.jad.2020.01.002

[11] Calder CN, Kwan ATH, Teopiz KM, Wong S, Rosenblat JD, Mansur RB, et al. Number needed to treat (NNT) for ketamine and esketamine in adults with treatment-resistant depression: A systematic review and meta-analysis. J Affect Disord. 2024;356:753–62. 38636712 10.1016/j.jad.2024.04.039

[12] Martinotti G, Vita A, Fagiolini A, Maina G, Bertolino A, Dell’Osso B, et al. Real-world experience of esketamine use to manage treatment-resistant depression: A multicentric study on safety and effectiveness (REAL-ESK study). J Affect Disord. 2022;319:646–54. 36167246 10.1016/j.jad.2022.09.043

[13] Rosso G, d’Andrea G, Barlati S, Di Nicola M, Andriola I, Marcatili M, et al. Esketamine treatment trajectory of patients with treatment-resistant depression in the mid and long-term run: Data from REAL-ESK study group. Curr Neuropharmacol. 2025;23(5):612–9. 39810448 10.2174/011570159X337670241029062524 PMC12163464

[14] Martinotti G, Dell’Osso B, Di Lorenzo G, Maina G, Bertolino A, Clerici M, et al. Treating bipolar depression with esketamine: Safety and effectiveness data from a naturalistic multicentric study on esketamine in bipolar versus unipolar treatment-resistant depression. Bipolar Disord. 2023;25(3):233–44. 36636839 10.1111/bdi.13296

[15] Chiappini S, d’Andrea G, De Filippis S, Di Nicola M, Andriola I, Bassetti R, et al. Esketamine in treatment-resistant depression patients comorbid with substance-use disorder: A viewpoint on its safety and effectiveness in a subsample of patients from the REAL-ESK study. Eur Neuropsychopharmacol. 2023;74:15–21. 37148637 10.1016/j.euroneuro.2023.04.011

[16] d’Andrea G, Miuli A, Pettorruso M, Cavallotto C, Marrangone C, Cocco , et al. Exploring vortioxetine combination with intranasal esketamine: A feasible alternative to SSRI/SNRI? - insights from the REAL-ESK study. J Affect Disord. 2024;367:583–8. 39233241 10.1016/j.jad.2024.09.004

[17] Turkoz I, Nelson JC, Wilkinson ST, Borentain S, Macaluso M, Trivedi MH, et al. Predictors of response and remission in patients with treatment-resistant depression: A post hoc pooled analysis of two acute trials of esketamine nasal spray. Psychiatry Res. 2023;323:115165. 37019044 10.1016/j.psychres.2023.115165

[18] Borentain S, Gogate J, Williamson D, Carmody T, Trivedi M, Jamieson C, et al. Montgomery-Åsberg depression rating scale factors in treatment-resistant depression at onset of treatment: Derivation, replication, and change over time during treatment with esketamine. Int J Methods Psychiatr Res. 2022;31(4):e1927. 35749277 10.1002/mpr.1927 PMC9720209

[19] Morrens J, Mathews M, Popova V, Borentain S, Rive B, Gonzalez Martin Moro B, et al. Use of clinical global impressions-severity (CGI-S) to assess response to antidepressant treatment in patients with treatment-resistant depression. Neuropsychiatr Dis Treat. 2022;18:1127–32. 35707064 10.2147/NDT.S358367 PMC9189368

[20] Clinical investigation of medicinal products in the treatment of depression - Scientific guideline | European Medicines Agency (EMA) [Internet]. 2013 [cité 20 juin 2025]. Disponible sur: https://www.ema.europa.eu/en/clinical-investigation-medicinal-products-treatment-depression-scientific-guideline.

[21] Fedgchin M, Trivedi M, Daly EJ, Melkote R, Lane R, Lim P, et al. Efficacy and safety of fixed-dose Esketamine nasal spray combined with a new Oral antidepressant in treatment-resistant depression: Results of a randomized, double-blind, active-controlled study (TRANSFORM-1). Int J Neuropsychopharmacol. 2019;22(10):616–30. 31290965 10.1093/ijnp/pyz039 PMC6822141

[22] Popova V, Daly EJ, Trivedi M, Cooper K, Lane R, Lim P, et al. Efficacy and safety of flexibly dosed Esketamine nasal spray combined with a newly initiated Oral antidepressant in treatment-resistant depression: A randomized double-blind active-controlled study. Am J Psychiatry. 2019;176(6):428–38. 31109201 10.1176/appi.ajp.2019.19020172

[23] Ochs-Ross R, Daly EJ, Zhang Y, Lane R, Lim P, Morrison RL, et al. Efficacy and safety of Esketamine nasal spray plus an Oral antidepressant in elderly patients with treatment-resistant depression—TRANSFORM-3. Am J Geriatr Psychiatry. 2020;28(2):121–41. 31734084 10.1016/j.jagp.2019.10.008

[24] Zaki N, Chen LN, Lane R, Doherty T, Drevets WC, Morrison RL, et al. Long-term safety and maintenance of response with esketamine nasal spray in participants with treatment-resistant depression: Interim results of the SUSTAIN-3 study. Neuropsychopharmacol. 2023;48(8):1225–33. 10.1038/s41386-023-01577-5 PMC10267177 37173512

[25] Daly EJ, Trivedi MH, Janik A, Li H, Zhang Y, Li X, et al. Efficacy of Esketamine nasal spray plus Oral antidepressant treatment for relapse prevention in patients with treatment-resistant depression: A randomized clinical trial. JAMA Psychiatry. 2019;76(9):893–903. 31166571 10.1001/jamapsychiatry.2019.1189 PMC6551577

[26] d’Andrea G, Mancusi G, Santovito MC, Marrangone C, Martino F, Santorelli M, et al. Investigating the role of maintenance TMS protocols for major depression: Systematic review and future perspectives for personalized interventions. J Pers Med. 2023;13(4):697. 37109083 10.3390/jpm13040697 PMC10141590

[27] Pettorruso M, d’Andrea G, Carlo FD, Risio LD, Zoratto F, Miuli A, et al. Comparing fast-acting interventions for treatment-resistant depression: An explorative study of accelerated HF-rTMS versus intranasal esketamine. Brain Stimul. 2023;16(4):1041–3. 37331507 10.1016/j.brs.2023.06.003

[28] Grabski M, Borissova A, Marsh B, Morgan CJA, Curran HV. Ketamine as a mental health treatment: Are acute psychoactive effects associated with outcomes? A systematic review. Behav Brain Res. 2020;392:112629. 32485203 10.1016/j.bbr.2020.112629

[29] Chen G, Chen L, Zhang Y, Li X, Lane R, Lim P, et al. Relationship between dissociation and antidepressant effects of Esketamine nasal spray in patients with treatment-resistant depression. Int J Neuropsychopharmacol. 2022;25(4):269–79. 35022754 10.1093/ijnp/pyab084 PMC9017766

[30] Mathai DS, Nayak SM, Yaden DB, Garcia-Romeu A. Reconsidering « dissociation » as a predictor of antidepressant efficacy for esketamine. Psychopharmacology. 2023;240(4):827–36. 36729145 10.1007/s00213-023-06324-8

[31] Echegaray MVF, Mello RP, Magnavita GM, Leal GC, Correia-Melo FS, Jesus-Nunes AP, et al. Does the intensity of dissociation predict antidepressant effects 24 hours after infusion of racemic ketamine or esketamine in treatment-resistant depression? A secondary analysis from a randomized controlled trial. Trends Psychiatry Psychother. 2025;47:e20220593. 37717263 10.47626/2237-6089-2022-0593 PMC12904269

[32] Vasiliu O. Esketamine for treatment-resistant depression: A review of clinical evidence (review). Exp Ther Med. 2023;25(3):111. 36793329 10.3892/etm.2023.11810 PMC9922941

[33] Estrade I, Petit AC, Sylvestre V, Danon M, Leroy S, Perrain R, et al. Early effects predict trajectories of response to esketamine in treatment-resistant depression. J Affect Disord. 2023;342:166–76. 37738705 10.1016/j.jad.2023.09.030

